# Formability of curved multilayer laminates via 3D printing using twisted continuous fiber composites

**DOI:** 10.1016/j.heliyon.2023.e20986

**Published:** 2023-10-13

**Authors:** Keigo Nakajima, Ryosuke Matsuzaki

**Affiliations:** Department of Mechanical Engineering, Tokyo University of Science, 2641 Yamazaki, Noda, Chiba, 278-8510, Japan

**Keywords:** Additive manufacturing, 3D printing, Carbon fiber, Continuous fiber

## Abstract

3D printers can print free-form 3D shapes; however, their mechanical properties are unsatisfactory. 3D printers can print 3D shapes freely but the resulting products exhibit unsatisfactory mechanical properties. 3D printing using CFRTP enables the formation of 3D structures with improved mechanical properties. When molding a structure with curved parts using a continuous carbon-fiber-reinforced thermoplastic (CFRTP) 3D printer, the difference in the inner and outer paths of the filament width during arc printing causes the CFRTP filament to become twisted, resulting in poor molding accuracy. In this study, we evaluated the formability of laminates via 3D printing with twisted CFRTP filaments to reduce the inner and outer path differences. And the maximum change in the filament width, which is defined as the maximum width minus the minimum width in one round of fibers, was defined as the forming accuracy. In the case of single-layer printing, the filament width decreased as the twist angle increased, and the forming accuracy (variation in the filament width) decreased. However, when stacking multiple layers, the maximum change in the filament width was the least when the twist angle was 6°. The discovery of the optimum twist angle at 1 K is the most significant aspect of this study and indicates the possibility of an optimum twist angle for various values of K.

## 1 Introduction

### 1.1 Limitations of CFRTP molding and molding method using 3D printer

Carbon fiber reinforced thermoplastic polymer (CFRTP), which uses thermoplastic resin, has received significant attention from the automotive and other industries [1] because it is easier to mold, mass-produce, and more recyclable than carbon fiber-reinforced thermoplastic polymer (CFRTS), which uses a thermosetting resin [2,3]. Consequently, CFRP becomes a composite material waste by the end of its life, and CFRP recycling has emerged as a challenge for scientists and engineers [[4], [5]]. Additionally, molding complex shapes into 3D shapes is challenging [6].

In recent years, the use of 3D printers as a versatile and low-cost technology for rapid casting and prototyping has increased in various research fields [7]. The application of this technology in diverse industries continues to expand, where the scalability of advanced 3D printers is exploited to create various materials with complex structures and shapes at the micrometer resolution [7]. Although 3D printers can print 3D shapes freely, the resulting product typically exhibit unsatisfactory mechanical properties. Compared with previous methods, 3D printing using CFRTP allows 3D structures with better mechanical properties to be molded.

### 1.2 CFRTP molding using 3D printer and the associated issues

Similar to automated fiber placement [8], the CFRTP filament injected during the curve printing of CFRTP is subject to waviness, twisting, and peeling from the bed, which can adversely affect the quality of the printed molded product [9]. When CFRTP is molded using an FDM 3D printer, unlike the results yielded by short-fiber or light-cured composite 3D printers, the mechanical properties of the molded product vary depending on various parameters such as the molding speed, number of fibers used, base resin, stacking pitch when stacking, and print pass [10].

Zhang et al. investigated the formation of manufacturing-induced fiber misalignment and breakage during the melt filament fabrication (FFF) 3D printing of 1 K continuous carbon fiber filaments. They showed that linearly printed stripe patterns featured high porosity and fiber misalignment, and that many printing defects such as path errors and fiber twisting occurred and worsened as the rotation angle and curvature increased because of excessive tensile forces from the nozzle [11]. Moldings printed using a CFRTP 3D printer (Mark Two, Markforged, MA, USA) indicate a gap between the print path and actual print [12]. The occurrence of the gap is analogous to the occurrence of a void in the area where the strength is improved by the fibers, and the fiber content in that area reduced, which resulted in a decrease in strength [13]. Shiratori et al. conducted a four-point bending test on an L-shaped specimen because no method has been proposed to evaluate the modulus of elasticity of CFRP with curved sections. First, compressive failure occurred at the outer surface of the curved section; the compressive failure stress decreased at an inner radius of 10 mm or less, and the elastic modulus decreased at an inner radius of 5 mm or less. The evaluation of the fiber orientation angle suggested that the decrease in the elastic modulus was caused by the fiber direction deviating from the circumferential direction owing to the twisting of the fiber bundle [14].

According to Sugiyama et al. the functional properties of the core material of a sandwich structure must be examined comprehensively and its shape must be optimized to maximize its characteristics; furthermore, the feasibility of forming a sandwich structure using a 3D printer than allows the arrangement of continuous carbon fibers should be assessed. The result showed that the twisting of fibers yielded defects, thus resulting in unsatisfactory precision and strength [15]. These studies showed that the challenge in molding CFRTPs using a 3D printer is the twisting of filaments, which resulted in reduced molding accuracy and mechanical properties. We believe that defect formation during curve printing is also affected by the difference in path tension between the inside and outside of the circle during arc printing.

### 1.3 Twist filament

The use of twisted filaments has been proposed to reduce the difference in path tension between the inside and outside of a circle during arc printing, which results in defect formation during curve printing. It is generally believed that twisting fibers for clothing improves the weaving performance compared with not twisting [16]. However, studies regarding the mechanical properties of carbon fiber multifilaments with twisting indicated that twisting at several tens of m^−1^ improved workability and weaving [17]. This suggests that the accuracy degradation caused by filaments that propagate back and forth continuously between the inside and outside of a CFRTP filament can be resolved by twisting the CFRTP filaments in 3D printed curve lamination.

In other words, when fabricating relatively large components, such as car components, the accuracy of CFRTP curvature printing becomes more important. The abovementioned points indicate that improving the accuracy of CFRTP when printing curved sections is an issue, and that the mechanism involved in the deterioration in accuracy when printing curved sections must be clarified. If the mechanism is clarified, then the printing path can be modified during curved printing, thereby resulting in improved accuracy. In addition, the strength is expected to improve significantly by reducing the gaps between the fibers and fiber breakage.

### 1.4 Proposal of current study

Based on the background provided in the previous section, this study proposes a curved multilayer stacked CFRTP 3D printing method using CFRTP filaments twisted in advance to improve the printing accuracy of curved fiber paths. Single-layer circular CFRTPs with twisted CFRTP filaments was molded, and the molding accuracy was evaluated based on the difference in twist angle and setting radius. Multilayer circular CFRTP 3D printing with twisted CFRTP filaments was performed, and the molding accuracy was evaluated based on the difference in the number of layers and twist angle. The results are summarized in Table 1.

**Table 1:** Printing conditions.

Head temperature [°C]	250
Bed temperature [°C]	65
Setting radius [mm]	5,10,15
Printing speed [mm/s]	2
Number of layers [−]	1,2,4,6

The improved forming accuracy of the twisted filaments enables the formation of more complex structures. In addition, the strength is expected to be enhanced significantly owing to fewer gaps between the fibers and fiber breakage.

## 2 Proposed method

### 2.1 CFRTP 3D printing

**Fig. 1:**
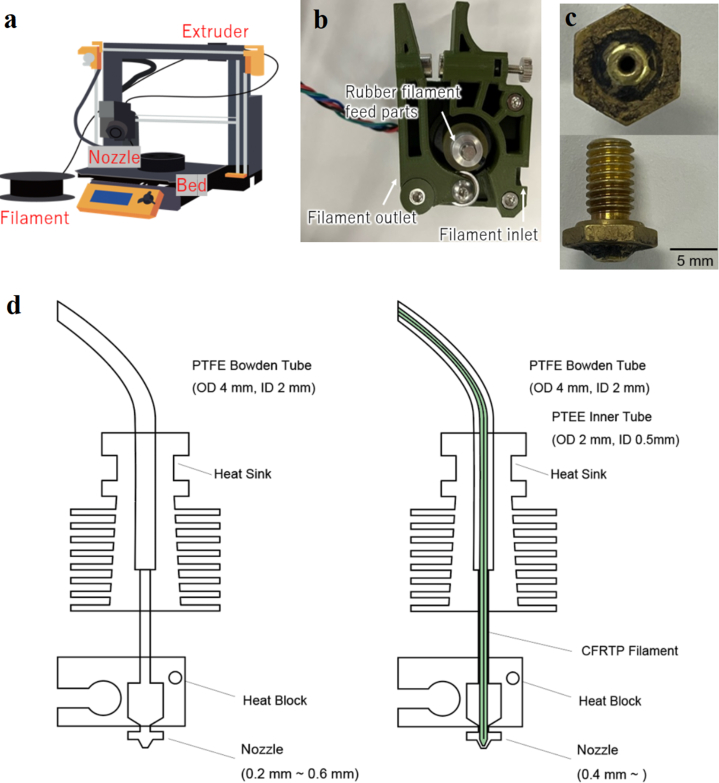
Overview diagram of Prusa 3 MK3S (Modified extruder and nozzle). **a**, Prusa 3 MK3S. **b**, Modified extruder for carbon fibers. **c**, Modified nozzle for carbon fibers. **d,** Improvement at hot end. (left: Before improvement; right: after improvement).

In this study, a 3D printer (Prusa Research, Prusa i3 MK3S), as shown in Fig. 1**a**, was used as the base. This 3D printer can be modified to 3D-print continuous carbon fiber composite materials or CFRTP. Specifically, the company developed its own G-code, modified the heater block and nozzle to use continuous carbon fibers, and modified the filament feed mechanism. To improve the hot end, a thin PTFE tube was inserted into a Bowden tube. The outer diameter was less than 2 mm to match that of the Bowden tube, and the inner diameter was at least 0.5 mm to match the diameter of the CFRP filament. And the extrusion volume of the firmware was consistent with the actual volume extruded by the extruder. The feed rate was corrected using firmware. The distance from the print start position to the arc position was determined by measuring the actual distance at which the bed and extruder were in stable contact. The drive gears and bearings in the extruder were modified to match the filament diameter. Thus, the filament can be extruded without it being sagged to a greater extent than its original condition.

In Prusa i3 MK3S, a G-code is written in an Excel file and saved in the TXT format. Finally, the 3D printer is connected to Repetier-Host, a 3D printing execution software, and the generated G-code is read to enable modeling on the 3D printer. The FDM 3D printer uses a stepping motor and pulleys attached to the extruder, which feeds the resin filament to the printer head at a constant speed. While the print head in the XYZ direction is controlled with a stepping motor, the heater block inside the print head melts the filament and pushes it out from the tip of the nozzle onto the hot table to laminate a three-dimensional shape. All the operations were controlled by the G-code. In this experiment, the code was rewritten to be compatible with CFRTP molding based on the G-code generated by Repetier-Host. The filament feed speed should be slowed to facilitate adhesion to the table, and the start position should be set away from the arc print path, as it is difficult for the filament to adhere to the table at the start of printing.

Normally, 1.75-mm-thick resin filaments are used for the FDM method, but thinner filaments were used in this experiment. Therefore, the filament feed mechanism needed to be modified. The filament used in this experiment had a diameter of 0.3 mm, and hence, it needed to accommodate this modification. By replacing the metal parts with rubber parts, we could accommodate the changes in the filament diameter (Fig. 1**b**). The nozzle was also changed, as shown in Fig. 1**c**, to match the filament diameter of 0.3 mm used in the experiment.

### 2.2 Twisted continuous fiber filament

**Fig. 2:**
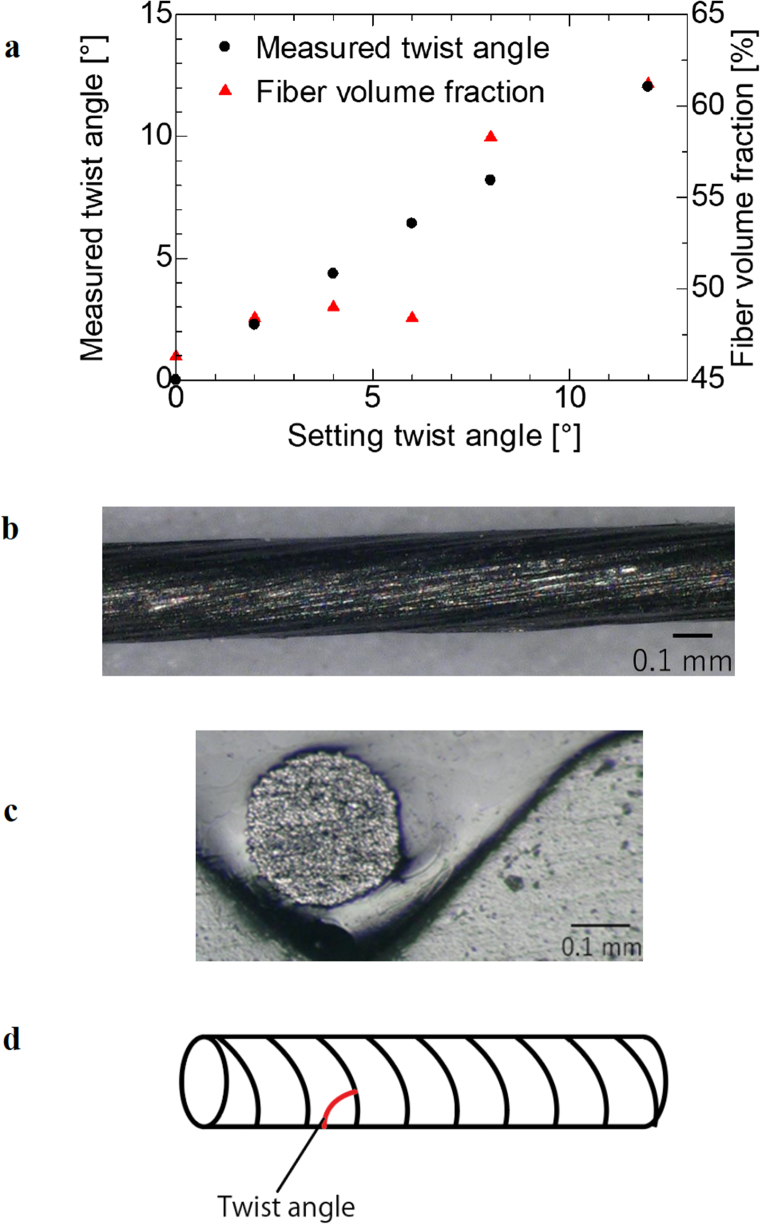
Twist filament used in the experiment. **a**, Specification of CF/PA6 twist filament. **b**, Photograph of twist filament. **c**, Cross section of filament with a twist angle of 6°. **d**, Definition of twist angle.

A twisted CFRTP filament manufactured by Kobe Steel, Ltd. was used as the filament under the conditions shown in Fig. 2**a**, where the left vertical axis is the measured value of the twist angle, the right vertical axis is the fiber volume content, and the horizontal axis is the set value of the twist angle (Fig. 2**b**). Carbon fibers and PA6 were used as the materials. For the twist angle, the red line is defined as the twist angle in Fig. 2**d**.

The printing parameters are listed in Table 1. The inner radius *r*_*i*_ and outer radius *r*_*o*_ of the circle were measured, and the average printing radius *r*_*p*_ was calculated. A digital microscope (Keyence, VHX-6000) was used to observe the cross-section of the specimens and to verify fiber breakage.

As with single-layer lamination, the inner radius *r*_*i*_ and outer radius *r*_*o*_ of the circle were measured for multilayer lamination, and the average printing radius *r*_*p*_ was calculated.

First, single-layer circular CFRTP 3D printing was performed using twisted filaments to evaluate the molding accuracy for different twist angles and setting radii. The same twist filament was then used to print a multilayer circular CFRTP to evaluate the effect of the number of laminations and twist angle on the molding accuracy and the difference in molding accuracy compared with that of the single-layer specimens.

## 3 Experimental results and discussion

### 3.1 Single-layer circular specimen

**Fig. 3:**
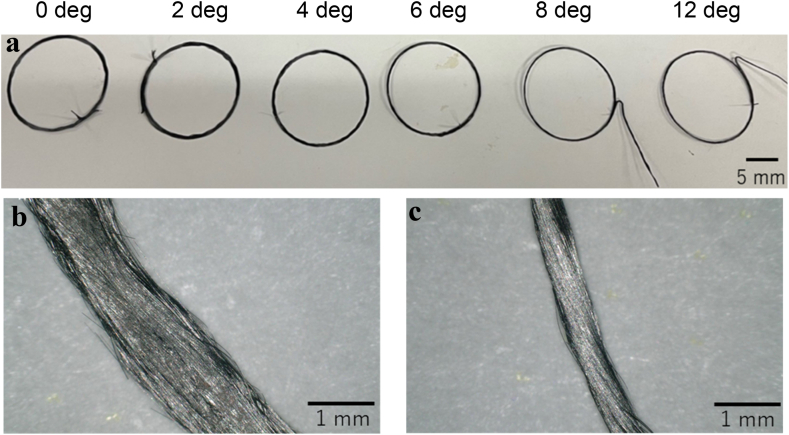
Photograph of a single-layer circular specimen printed with a setting radius of 15 mm. **a**, Overview of the specimen. **b**, Enlarged photograph of the specimen printed with a twist angle of 0°. **c**, Enlarged photograph of the specimen printed with a twist angle of 0°.

**Fig. 4:**
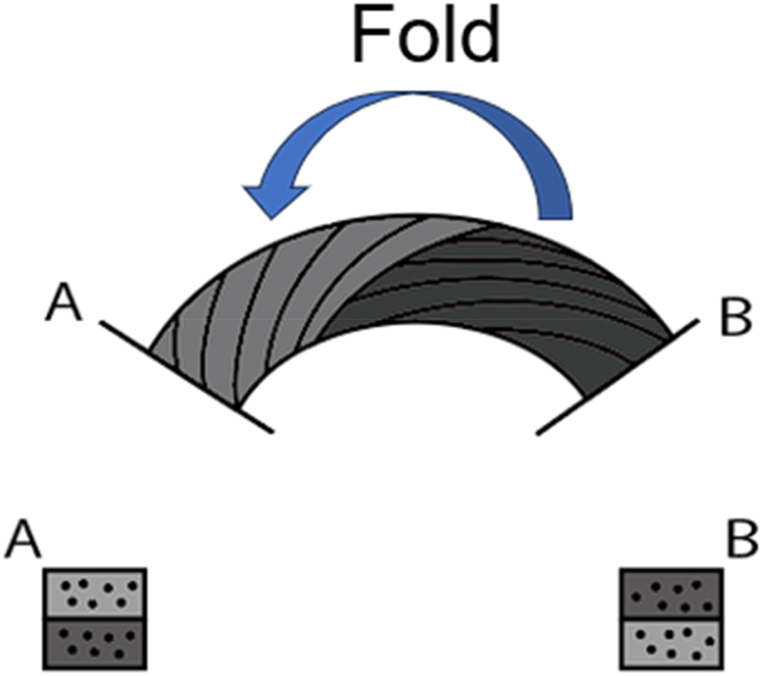
Schematic of fold back.

Single-layer circular specimens were printed with the radii of 5, 10, and 15 mm and observed using a digital microscope (Keyence, VHX-6000). The specimen with the radius of 15 mm is shown in Fig. 3**a**, and it was observed using a digital microscope as shown in Fig. 3**b** and **c**. The observation of the printed material revealed that the fibers on the bottom and top surfaces folded back and forth as if they were interchanged. When printed on a 3D printer, the nozzle does not rotate, thus resulting in a wrap-around motion for every half-lap, as shown in Fig. 4. The filament width of the curved section tended to decrease as the twist angle increased, and the variation in the filament width of the curved section also tended to decrease as the twist angle increased.

**Fig. 5:**
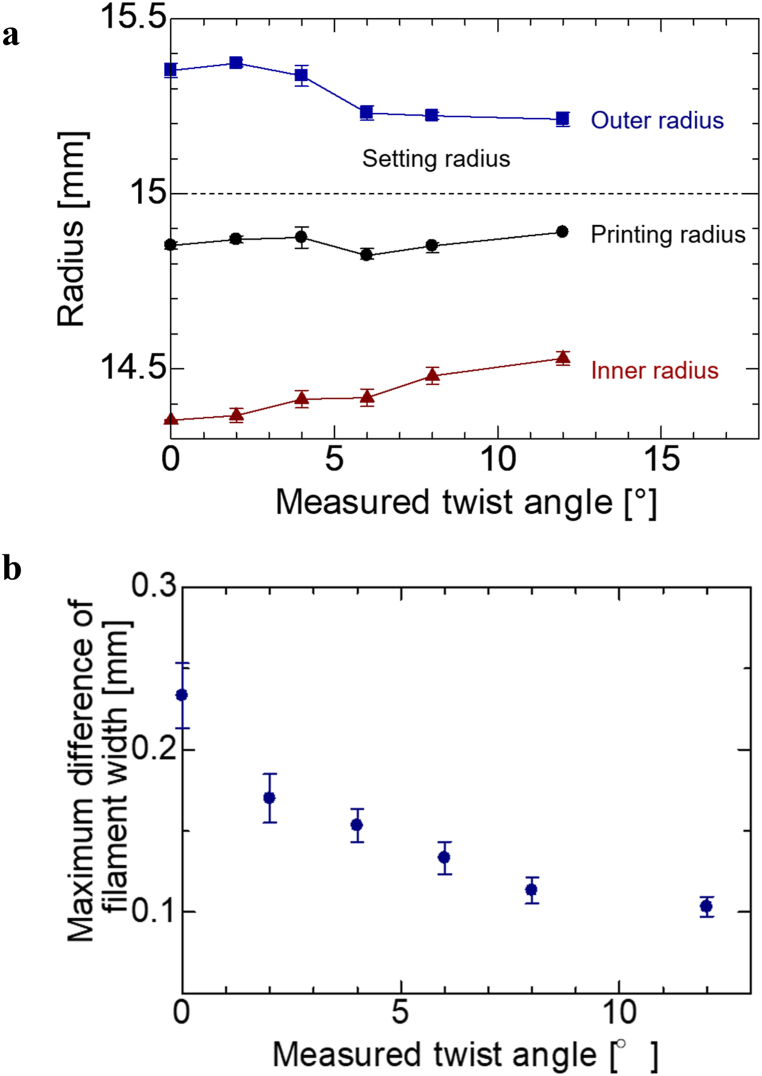
Relationship between the twist angle and various parameters at the setting radius of 15 mm. **a**, Relationship between the radius and the twist angle at the setting radius of 15 mm **b**, Relationship between the maximum difference in filament width and the twist angle at the setting radius of 15 mm.

The inner and outer radii were measured four times each for the specimens with the radii of 5, 10, and 15 mm, and the average of the two values was calculated as the print radius. As a representative example, a graph for the specimen with a print radius of 15 mm is shown in Fig. 5**a**. The calculated results show that, regardless of the twist angle, the printing radii were approximately 4.9 mm, 9.8 mm, and 14.8 mm when the setting radii were 5 mm, 10 mm, and 15 mm, respectively. The print radius was smaller than the set radius, which was confirmed by Matsuzaki et al. [12], and we consider this to be the correct result. There was a tendency for the printing radius to approach the setting radius as the twist angle (twist amount) increased. When the twist angle was large, the filament width was small and the fiber bundle was not unraveled. This is believed to be because, when the fibers are twisted more, the effect of further twists is smaller.

The maximum change in filament width was defined as the maximum width minus the minimum width in a single fiber lap, and the relationship between the twist angle and the maximum change in filament width was evaluated. As a representative example, a graph for the specimen with a print radius of 15 mm is shown in Fig. 5**b**. The graph shows that, as the twist angle increases, the variation in the filament width decreases, which indicates that the forming accuracy is improved. The twisting of the fibers renders them less susceptible to folding. This prevents fiber breakage during folding, thereby reducing fiber scattering.

### 3.2 Multiple circular specimens

**Fig. 6:**
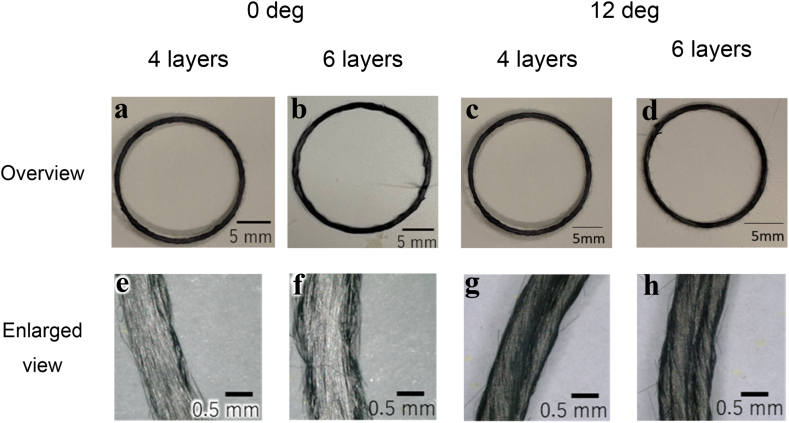
External view and magnified images of circular specimens printed at 0° and 12° twist angles.

The printing results of a multilayer circular specimen printed with a setting radius of 15 mm are shown in the photographs in Fig. 6**a**–**d**. The filament width at the curve of the specimen tended to increase as the number of layers increased. Fig. 6**e**–**h** shows the results of the observation of each printed specimen using a digital microscope (Keyence, VHX-6000). The filament width at the curve of the specimen tended to increase as the number of layers increased.

**Fig. 7:**
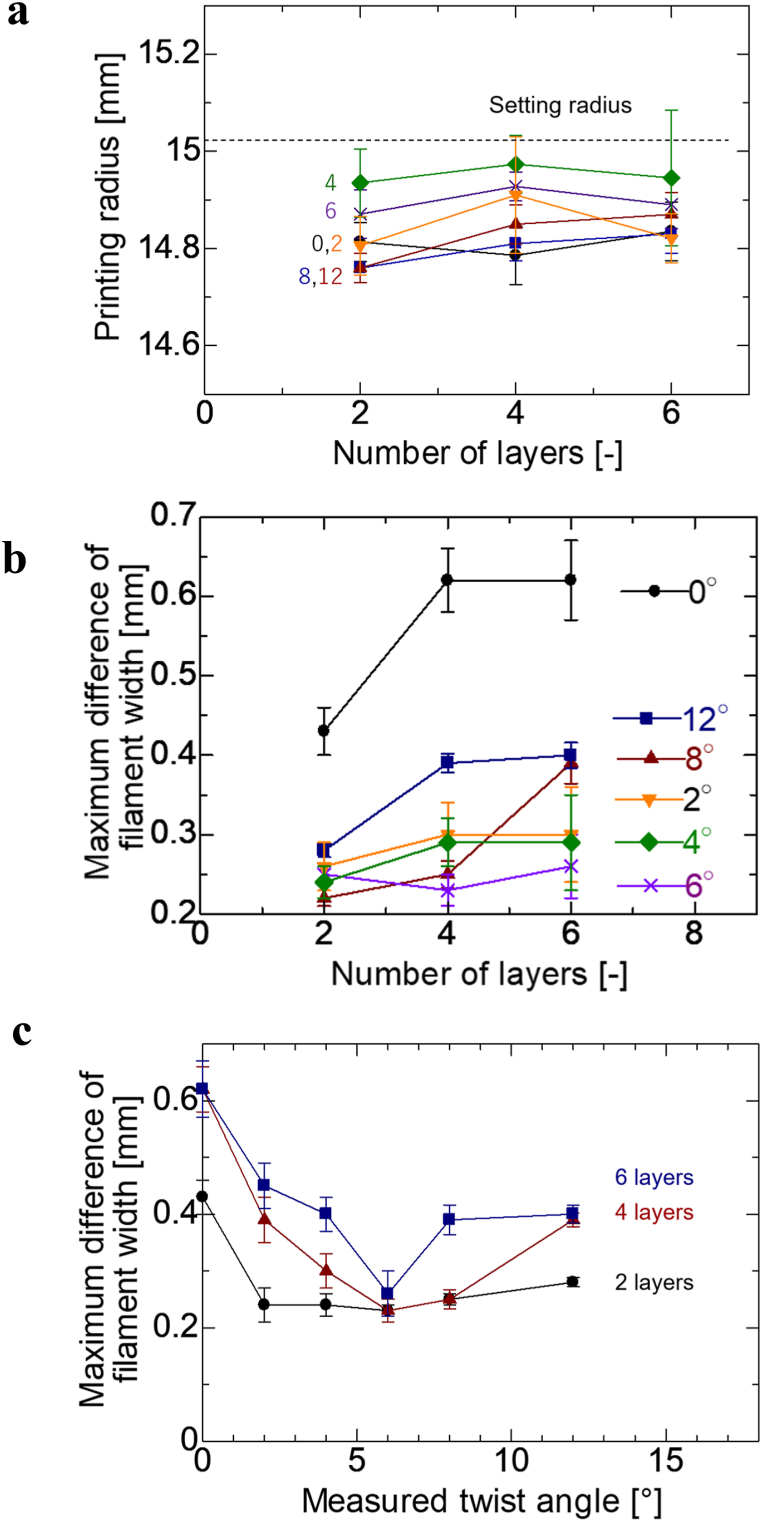
**A** Graphical representation of relationship between printing radius and number of layers in multilayer printing for each twist angle. The graph shows the relationship between the printing radius and number of layers in multilayer printing. **b** Graphical representation of relationship between maximum difference in filament width and number of layers for each twist angle in multilayer printing. **c** Relationship between maximum difference in filament width and twist angle in multilayer printing for each number of layers.

Fig. 7**a** shows the printing radius and twist angle on the vertical and horizontal axes, respectively; as shown in the figure, the inner and outer radii vary with the twist angle. This is believed to be because the effect of misalignment between layers is greater than the effect of twisting the filaments, which causes the fibers to be twisted once more. The number of layers of the specimen filaments produced is represented on the horizontal axis, whereas the maximum change in the filament width is represented on the vertical axis, as shown graphically in Fig. 7**b**. The maximum change in the filament width on the vertical axis and the twist angle on the horizontal axis are shown graphically in Fig. 7**c**. Fig. 7**b** shows that the maximum change in filament width increased as the number of layers increased. As the number of laminations increases, width variations due to misalignments to the laminations are more likely to occur. Consequently, the filament width of the specimen varies more significantly. Fig. 7**c** shows that the maximum change in the filament width was the smallest for any number of layers when the twist angle was 6°. This indicates that a larger twist angle does not necessarily lead to a higher forming accuracy, as in the case of a single layer.

**Fig. 8:**
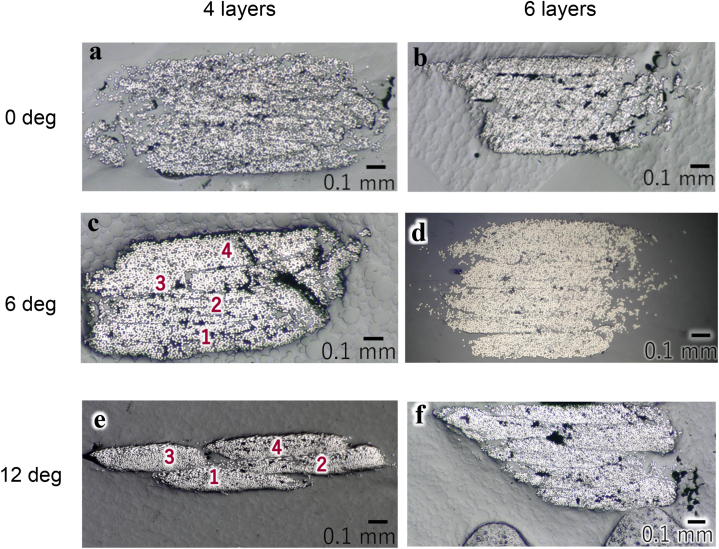
Cross-sectional photographs of circular specimens in multilayer lamination printing with varying twist angles and numbers of layers (The numbers are in lamination order.).

To determine the reason why the maximum change was the smallest at the twist angle of 6°, as shown in Fig. 6, cross-sectional photographs of the specimens were taken at the twist angles of 0°, 6°, and 12° (Fig. 8). The deviation between the layers was small at the twist angle of 6°. When the twist angle was small, the fibers were unraveled owing to the twisting effect, resulting in misalignment between the layers. When the twist angle was large, each layer was too thin to be laminated and the adhesion between the layers was weak, resulting in misalignment between the layers. The experimental results showed that twisting can reduce the variation in the filaments and may result in structures that could not be achieved using previous methods. Additionally, the optimal twist angles may exist for various fiber bundles. However, the mechanical properties have not yet been evaluated; therefore, further studies are required to demonstrate the improvement in the mechanical properties resulting from improved formability.

## 4 Conclusion

The forming accuracy of continuous twisted fiber composites during curve-layered printing was evaluated by investigating the relationship between the number of curved layers and the forming accuracy (variation). The results of this study are as follows.

● The molding of circular CFRTP was performed using CFRTP filaments, and differences in molding accuracy owing to the differences in twist angle and radius were evaluated.
● For single-layer printing, the forming accuracy improved when the twist angle was large. This is because the fiber is less likely to be twisted when the twist angle is large, which prevents fiber breakage and reduces the variation in the filament width.
● In the case of multiple layers, the maximum variation in the filament width in the curved section was the least when the twist angle was 6°. When the twist angle was small, fiber breakage occurred because of the twisting effect of the fibers, which resulted in a variation in the filament width. Consequently, the lamination was misaligned. When the twist angle was high, the variation in the filament width reduced; however, because each layer was thin, the variation was insignificant and the adhesion between the layers was weak, which resulted in misalignment between the layers.

We considered that the smaller the variation in filament width, the closer the forming to the set path. The discovery of the optimum twist angle at 1K is the most significant aspect of this study and indicates the possibility of an optimum twist angle for various values of K. However, as the number of layers increased, the effect of misalignment between the layers become more prominent, and improving the forming accuracy using only twist filaments was difficult. The purpose of this study is to evaluate the effect of twisting on the forming accuracy of filaments; strength evaluation is not included in the scope of this study because it is not directly related to the evaluation. In the future, we plan to evaluate their strength by performing tensile tests.

## Data availability

The raw/processed data required to reproduce these findings cannot be shared at this time as the data also forms part of an ongoing study.

## Additional information

No additional information is available for this paper.

## Declaration of competing interest

The authors declare that they have no known competing financial interests or personal relationships that could have appeared to influence the work reported in this paper.
